# Researches, Critical and Experimental, upon the Capillary Circulation

**Published:** 1849-04

**Authors:** Bennet Dowler

**Affiliations:** New Orleans. Corresponding Member of the Academy of Natural Sciences of Philadelphia


ART. IV.—*Researches, Critical and Experimental, upon the Capillary Circulation.* By BENNET DOWLER, M. D., of New Orleans. Corresponding Member of the Academy of Natural Sciences of Philadelphia.

The composition of forces involved in the circulation of the blood, is among the subjects of physiology still shrouded in obscurity. Few topics, since the discovery of Harvey, now more than two hundred years ago, have occupied the attention of physiologists more than this, and the fact that it still affords an ample field for discussion, and hypotheses, is conclusive evidence that it involves problems not easily solved. It illustrates, indeed, in a striking manner, the intricacy and difficulty of the investigations of processes going on within the living organism.

As regards the circulation in the order of vessels situated between the termination of the arterial, and the commencement of the venous systems —the capillary system—our knowledge is especially defective. That there are forces incident to the capillary circulation, independent of the action of the heart, and which are capable of exerting considerable influence over the entire circulation, is, we think, apparent. The amount of blood which an organ, or structure, receives, is by no means determined solely by the action of the heart, and the calibre of the distributing vessels, but involves, in addition to these, the physiological condition of the organ, or structure, itself. Under different physiological conditions, an organ, or structure, will claim, and receive, different quantities of the circulating fluid.

For example, the gravid uterus contains vastly more blood than before impregnation, and this change, it is obvious, does not follow from a change in the general forces carrying on the arterial circulation, nor in a prior increase in dimensions of the uterine arteries. It proceeds from an increased force developed in the organ itself.

To understand the causes of the capillary circulation better than we do, is not alone desirable in a physiological point of view. The subject has very important pathological relations. It is connected more especially with the ultimate nature of the morbid condition which we call *inflammation*. The first, and most characteristic of the appreciable events belonging to the inflammatory state, is an increased supply of blood to the inflamed part, and this supply proceeds, evidently, from a morbidly developed attraction for blood in the inflamed part. Most of the sensible phenomena of inflammation, such as swelling, heat, redness, are due to the accumulation of an increased quantity of blood. They are but secondary results of that unknown dynamic, or chemico-vital perversion which gives rise to a greater afflux of blood to the inflamed part than obtains in health. Now, if we were able to appreciate the dynamic or chemico-vital forces which preside over the normal capillary circulation, we have every reason to conclude that the true character of that pathological condition which involves an excess, or perversion of these forces, would be intelligible.

Nor is it likely that the pathological applications of a more intimate acquaintance with the physiology of the capillary system would be limited to a single morbid condition. All local diseases, involve, to a greater or less extent, aberrations of function in this order of vessels, and all would doubtless be better understood in proportion to the advance of physiological knowledge in this respect.

These few remarks are made by way of introduction to some extracts from a paper which appeared in the last number of the New Orleans Med. and Surg. Journal, from the pen of Bennet Dowler, M. D., the title to which is prefixed to this article. We do not intend to discuss any of the hypotheses that have been submitted on the subject to which the paper relates, nor to undertake any critical analysis of the latter. We have been much interested in the perusal of the essay by Dr. D., and his experimental researches on this subject are, to say the least, novel and curious. Under the impression that our readers will participate in the interest which we have experienced, we shall proceed, without farther comment, to make two quotations, which will embrace the larger portion of the entire paper.

As the title states, the researches are *critical* and *experimental.* From the portion of the paper which is occupied with criticisms upon several of the hypotheses more or less in favor with physiologists at the present time, we select his remarks upon *microscropical investigations.* We select these remarks, because they are quite at variance with the views now entertained by some of the most eminent physiological observers and writers, and it will be perceived that the objections of the author to the validity of appearances disclosed by the microscope, not only apply to inquiries respecting the capillary circulation, but, also, to other branches of physiology upon which that method of study is supposed by many to have already shed not a little light, and which promises still further revelations of importance. As respects the soundness of the considerations set forth in the following extract, we will not offer an opinion:

"As the capillary system belongs to Microscopic Anatomy, it will be necessary to dwell a few moments upon this aspect of the subject—a necessity, that I regret, not because my observations have been few, but for a different reason, namely, an extended monograph would be required to place my views on this subject in such a light, as would enable me to indulge a reasonable expectation of escaping the charge of presumption, in dissenting from the highest authorities, not, indeed, so much with respect to the exact structure of the capillary vessels, (which is little known) but, in respect to other elementary points still more uncertain. For example, the globules of the blood, so often minutely described, pictured, classified, (as belonging to different animals) nay, absolutely anatomized, are probably, to a great extent, illusory. Air, or rather carbonic acid gas, so abundant in the blood, with no other anatomical membrane than that which envelope a soap or champaigne bubble, would appear like globules—or these supposed blood globules may be heterogeneous liquids, having different densities, different affinities for each other, for the serum of the blood and for the surrounding tissues. Hence, upon the physical principle of homogenous attraction, the albumen, the fibrin, or the oily matters in the blood, would, like oil in water, assume the globular form. Besides, the microscopic analysis in the direct light of the sun, gives to blood and certain vascular tissues, a wholly new, different, and unvaried appearance. Of this method, I have much to say, but not on this occasion. I wish now, to be understood as referring to the usual degree of light, though unfortunately this important point remains without any standard of comparison, except the negative one, requiring the light of a lamp, or the indirect light of the sun. Passing by this source of illusion, the reader will be kind enough to read the following extracts, taken almost at random from a few books, lying on my table,—more would be tedious.

"Prof. H. Milne Edwards, represents the globule opened, dissected, and the flaps turned back! (*Anat. and Phys.* 25.) A microscopic object, would, of course, require a microscopic dissector, and microscopic scalpels! Dr. Golding Bird, in his account of Prof. Mulder's chemical physiology of the red coloring matter of the blood, writes with apparent good faith, many passages like the following:

'The coloring matter is normally enclosed in a sac or cell composed of a thin membrane, consisting chiefly of protein, and capable of admitting of percolation through its walls under certain circumstances, although it generally retains the colored fluid it encloses sufficiently firmly to prevent its admixture with the serum. Within the sac is a nucleus of similar chemical composition to the membrane, and the whole thus organized, constitutes the *blood-disk*, *globule*, or *red particle*. The bright red corpuscules are always *biconcave:* in the dark-red blood, the corpuscles are *convex*, and the enveloping membrane is *much thinner;* they readily burst, produce exosmosis, emptying the sack, &c., &c.: On reaching the capillaries, the coating of oxyprotein is removed, the protein being employed for the repair of tissue, and the oxygen used for effecting the metamorphosis of effete and exhausted structures. The corpuscles losing their opaque covering, have their power of reflecting light diminished, their concave surfaces are lost, and the whole assumes a venous tint." (N. Y. Jour. Med., and Lond. Med. Gazette, 1845.)

Mr. G. Newport, in a paper read before the Royal Society, undertakes to define the corpuscles of insects, and other invertebrata, comparing them with those of man, and the vertebrata: He says "in the articulata the forms are first, the *molecules;* secondly, the *nucleated* or *oat-shaped corpuscle;* thirdly, the *spherules*, or minute rounded bodies developed from the oat-shaped corpuscle, analagous to the free nucleoli of Valentin; and lastly, the *discs*, which are further developements of the spherules, and analogous to the true red-blood-discs of the higher animals.'—(Bulletin of Medical Sciences. 1845.)

'No single description (of the red globules of the blood) has tallied with that which went before. Leeuwenhoeck believed that he saw them consisting each of *six well compacted, smaller globules.* Hewson believed that they were *bladders* which had within them *some* central body, loose and moveable; that often the central part might be seen *rolling* in its bag, &c. The Abbé Torre, said that these were not globules, but *rings.*'—(Bell's Anatomy and Physics.)

Dr. Carpenter calls the *globules*, '*flattened* discs, having a circular form.' —(Phys. § 570.)

Sir Charles Bell relies on the microscopic examination of the frogs foot, for the proof of the capillary vessels. He says naively enough:—'We do not discover the coats of the vessels, but conclude that they exist, from the confined and certain course of the particles (of blood) which are in motion.'—(Anat. ii. 26.)

"Micography envisages much which seems
"For man's illusion given."

Thus a convex or a concave surface, may, under certain circumstances, appear globular. The different elements of the blood, as air, water, fibrin, fatty matter, albumen, not to mention their probable form, possessing, as they do, different degrees of transparency, different densities, different degrees of shading power, of irritation, and more than all, different degrees of refracting force, would all conspire to throw doubt upon the anatomical accuracy of these microscopic membranes, and central nuclei, and the configurations which are too confidently proclaimed as absolutely characterizing the globules. Still more. Physical capillarity itself, would be illusory, that is to say, a portion of liquid, or several liquids in a capillary tube, would assume a concave or convex form, according to their wetting or non-wetting properties. These, and some similar facts, were forced on my attention, in making dried preparations between narrow strips or plates of glass; pressure and desiccation for a whole season, did not deprive many preparations of these globules of air, water, &c. Heat, instead of expelling them, often augmented their number, by expansion, at the same time giving fixity to their enveloping tissues.

I might adduce still farther arguments from dead or physical chemistry, favorable to the doctrine here advanced, as to the globules of the blood being in all probability nothing more than one or several of the constituents of the blood taking the spherical form, as may be observed, out of the living body, without an organization high and complicated. I have observed with the naked eye, in the recently dead human subject, a rapid circulation or flow of chyle in the blood; the former had not yet fully mixed or combined with the latter, as will be seen in the sequel. Hence, this element alone, might be expected to assume the form of globules, without admitting anatomical sacks, nuclei, corpuscles, &c. The very pictnres of these condemn them, on the physical principles of light. The centre is said and is known to be colorless, nay, luminous, if the globule be organized with red envelopes, the rays would be absorbed to a great extent, while upon the supposition that it is simply, like a drop of water, or oil, or like a double convex lens, it would converge the light, so as to give a central luminosity corresponding with the pictorial representations. This piece of anatomy, this complete and sudden organization of the blood globule, considering the rapidity with which it is destined to pass through the minutest tubes, is, in a teleological point of view, very improbable, and must be, upon the filtering, wetting, or endosmotic theory of the circulation, somewhat unfavorable to permeation.

I do not deny that there are globules in the blood, but I deny that they are peculiar to it, since they appear to exist equally in every animal and vegetable substance which I have examined. But of all objects, they are the least likely to appear truly represented in the microscope either as to figure, color, or anatomical organization.

Color is an unimportant, and an exceedingly variable, not to say a deceptive element, in microscopic research. A very thin stratum of blood absorbs little, transmits much, and reflects almost no light in the direct solar ray. The so called red globules, may be absolutely colorless, though coloring matter near them, or in the axial line of vision, gives them a red hue, in the same way that a red curtain, in a room with a mild light, colors microscopic objects red? Are the peaks of an elevated mountain chain really *blue?* the clouds that shimmer in an autumnal setting sun, really *red?* a full moon in Indian summer, *bloody?* Are solar and lunar coronæ and halos, and the rainbow reflected and refracted from falling globules of rain, *real colorations?* The tissues and their enclosed liquids, differing in density, in figure, and in diaphaneity, might be expected, themselves, in some instances, according to position, to magnify like the microscope, and to decompose light like prisms, or falling rain drops, while on the other hand, absolute transparency in the object, renders it as invisible, and as structureless, as the air itself. Moreover, a dim light, at certain angles, throws enormously long, or very short shadows. One object rises above another, while the local point or field of vision, is always, if accurate, extremely limited; and, hence, diagrams and descriptions, however correct, explain little, where the thing itself is involved in an almost inextricable confusion, whether the fluids, or their containing vessels, be the object of research. For example, in the examination of fluids, I have observed a very striking, possible source of fallacy, namely, an apparently spontaneous formation of very regular capillaries, resulting from the desiccation of animal fluids. During this process, air, water, or some portion of the liquid, perhaps influenced by homogenous attraction, arranges itself at first, into river shaped channels, or tubes, with many branches, all of which contract more until they become fixed, while innumerable anastomosing connections join with other similar trunks and twigs, forming a net-work very like capillaries, though more open, and straight. Other fluids, in dessicating, form innumerable fissures, different, it is true, from the above, and from capillaries, but the tracks of these fissures correspond very closely to some portions of the net-work of capillaries, and may be even mistaken for them. Before I ascertained the fact, first mentioned above, I had concluded, erroneously, as to the quality of mucus expectorated by a patient. I found, as I supposed, capillary vessels under the microscope. There is, as I accidentally noticed, another possible source of error, in making capillary preparations between plates of glass, which though closely bound together, and kept in box, permitted, nevertheless, the formation of cryptogamous plants, (chiefly *Fungi*) of several kinds invisible to the naked eye, but exceedingly beautiful in the microscope, having delicate stems, which by crossing each other's path, seemed to form a capillary net-work, among the tissues, and in other places. Their radicles and trunks, though extremely minute, are much more linear and uniform in size than capillaries.

I have examined hundreds of capillary specimens, both human and comparative, both wet and dry, healthy and morbid;—in the capsule of the lens, the hyaloid membrane, the iris, the retina, the choroid, the pia mater, tunica vasculosum, the serous, mucous, and other tissues in the principal structures of the human fœtus as early as the second month; also in the beautiful, and highly transparent tissues of young alligators, as the nictating membrane, retina, hyaloid, iris, mesentery, peritoneum, etc. But, *a priori* conclusions and conceptions were not realized; capillaries like regular rivers growing from thousands of tributaries—capillaries like arteries and veins, with thousands of ramifications, forming a regular and continuous net-work, were not seen with that uniformity, as to enable me (whatever others may have seen,) to draw a satisfactory conclusion, where a capillary vessel begins, where it ends, and what is its common direction. Isolated points, centres or reservoirs, are sometimes seen, from which capillaries radiate as if wholly independent, as if possessed of circulations within themselves. Again, a large, short canal, a spongy islet, or a stellated point, receives small vessels without any apparent outlet! The same may be affirmed of many regular arborizations, which seem as isolated as one spider's web is from another's. The capillaries, sometimes, appear rough, porous, and spongy;—conditions unfavorable to the perpetuation of motion generated in the heart. They enlarge and diminish irregularly—tap each other at every possible angle—form every possible curve, if not every possible figure.

"From the result of microscopic examinations, little doubt rests on my mind," says Prof. Jackson, of Philadelphia, "that a large proportion of what is regarded as capillary circulation, is not in fact performed by vessels. On the contrary the blood circulates out of vessels, but in currents. The currents of globules flow in every direction; I have seen currents of globules commence when none existed—pursuing every course with great diversity in their velocity, etc.—some in a retrogade course." (Princip. Med. 23.)

The importance of the microscope, cannot easily be overrated, in the revelation of minute objects, showing their outlines, organs, general conformation, and the *tout ensemble*, all of which are very striking in a moderate light; but, unfortunately, it fails to a great extent, exactly where, judging *a priori*, it ought to afford the most satisfactory results: for, in many instances, instead of drawing more clearly the lines of distinction—instead of showing, in a striking manner, the specific differences between tissue and tissue, between fluid and fluid, it reveals a closer similarity, a nearer analogy, if not identity. Thus when properly prepared, the fibres of muscles fasciæ, tendon, pleura, peritoneum, arachnoid, sclerotica, etc., will appear so much alike, that the microscopist will often have to look at the labels to tell the difference: at least such is the case as to myself, and it is still more so to some of my friends. He who has a good eye, will probably distinguish certain objects, with it, better than with the microscope, as the difference between a muscular and nervous fibre, a nervous and arterial twig, etc.; whereas, the contrary rule, holds good as to infusorial organizations, and multitudinous objects of a minute size, and of sufficient diapheneity.

It appears to me quite impossible, to see, and describe, with anatomical accuracy, the capillary vessels, the circulation of globules, and their structure, or even the groups of organs in many objects called microscopic, not to mention entire animals, as large insects, the limbs of frogs, etc. Even the most delicate and transparent infusorium, is a great mass of tissues, piled one above another, presenting different results, at different perpendicular foci, and giving different appearances, with the increment or decrement of light, etc. Suppose that an entire frog, or man, in a transparent state, were placed in a microscope; millions of vessels will be found in the same field of vision, or in the same axial line, the one above the other, traversing each other in every direction; every change of glasses will present a new focus and a new phase, *ad infinitum.* It is probable that a life-time spent in this confused method of examination, would produce no satisfactory anatomical result. Suppose a thin layer of tissue is to be examined. Here, the chances of error are diminished, but they still exist—an object is selected, as a capillary. Is it a tube, a muscular fibre, or something else? For it is difficult to determine in the microscope, a solid cylinder from a tube. But, suppose a tube is found isolated, not one among many in the axis of vision,—is it a true capillary, a vein, a lymphatic, or an artery? How can its structure or coats be described, as some pretend to describe them, without first laying open this tube, in order to examine its different strata, upon an exact level? Who can do this, in an object invisible to the naked eye? It is not the experimenter, but the reader, with pictorial illustrations before him, that has clear conceptions of these things. Indeed, Schiller contended that the Ideal was always better than the Real. Thus, of morality and liberty, he says,—(he might have included Micography):

——— ——— ——— If we test
By the *idea* of the good, the best,
How mean our efforts and our actions are!
* * * * * * *
Freedom is *only* in the land of *dreams*
And only blooms the beautiful in song.

The hesitating manner and the contradictory accounts of different authors, show that the anatomy of the capillary system is but little known. The micographers of the seventeenth and eighteenth centuries, investigated this subject with great industry and ability, and it is fair to presume that this part of anatomy is attended with great difficulties, since so little has been clearly established. Never were results more sterile, notwithstanding the grandiloquence of the day.

Beclard says, that "minute injections and microscopical observations soon lead anatomists to admit that instead of the supposed parenchyma interposed between the terminations of the arteries and veins, according to the ancients, everything in the body is composed of vessels; an opinion which yet divides all the cultivators of the science." "The capillaries are intermediate between the arteries and veins. It is in these vessels that, insensibly and without any fixed point, the arteries are converted into veins. However, the capillary vessels have been generally described as the last division of the arteries, rather than the beginning of the veins. Whether this be well founded, etc. the texture of the capillary vessels cannot be observed with the naked eye. These vessels have very thin and soft parietes, invisible to the naked eye, and slightly visible with the microscope, very little different from the substance of organs, and also from the humors they convey. They seem rather formed out of the substance of organs than provided with its (their) own parietes." It would be impossible to stick the point of a fine needle without opening several of these vessels. Beclard thinks it quite impossible to decide upon the existence of the colorless or serous capillaries on which much has been written. He adds,—"Doellinger thinks that the arteries, at their extremities, cease to have any parietes, and that the blood flows unconfined in the solid substance of the body. Wilbrand goes still further: asserting that the whole of the blood is converted into organs, etc."

Professor Carpenter's anatomical history of the capillaries does not seem embarrassed with any doubts whatever, though it bears on its face very little that can be called absolute certainty. He says that "the capillary circulation is carried on through tubes which have *distinct membranous parietes;*—originating in cells"!—in another place, he says that "the capillaries *arise* from a minutely anastomosing *net-work*, into which the blood is brought by the ramifications of the arteries on *one* side, and from which it is returned by the radicles of the veins on *the other*." (Physiol. 1848.) Cells! net-work! ramifications! radicles! one side! and the other side!

The Medico-Chirurgical Review, unwilling to allow the capillary system any share in vital phenomena, espouses "the Cell-Theory, as the greatest of all physiological discoveries! Every animal and plant is developed from a *nucleated cell*, by which all the organic tissues are formed, and by which nutrition, secretion, absorption, assimilation, growth, decay, etc., take place; all these processes are *independent* of *the blood-vessels*, being *extra-vascular*, as all accurate observers in every part of Europe testify." (See New Orleans Med. and Surg. Journal for 1844–5)

The micographical physiologists are now making great efforts to plant the tree of life in a "*cell!*"

A better type of life can be found in an animal never before described which, I have called, *the solar infusorium*, (because discernible only in the direct ray of the sun,) entering, as it does, like bricks into the cell wall, and into every other tissue. A Nilotic crocodile, supposed to have been dead five thousand years, is, to a considerable extent, composed of this animal, which springs into life, as soon as the tissue is sufficiently dissolved. I am fully able to show that the motion of this animal is not owing to evaporation, which, as I suppose, renders this discovery complete. This animalcule is very distinctly seen with a moderate magnifying power—a high power is too inaccurate. It is probable that it cannot be killed, certainly not by boiling, and the strongest heat short of combustion. (The latter, perhaps, only dissipates it.) These animalcules are all exactly alike. The *Vibriones* of Ehrenberg, Mandl, Mantell, and others, approximate the *solar infusoria*, but are essentially different, taking the descriptions and figures of these authors for a guide. The solar infusoria enter into, and constitute the chief material of the different tissues of every *infusorium* that I have examined. In all hard bodies they are, of course, motionless, until solution be effected. Whether they are the *ultimata* of organic life, is uncertain:

> "Great fleas have lesser fleas, and these were made to bite them,
> And these fleas have lesser fleas, and so on *ad infinitum*."

In the quotation which follows, the reader is presented with nearly all of the portion of the essay which contains the *experimental* researches, together with the author's comments thereupon. The observations are certainly curious, and, so far as we know, original. They appear to demonstrate conculsively the independence of the capillary circulation, and, also, that this circulation does not cease immediately after death. The interest of the observations relates chiefly to the latter fact, inasmuch as the existence of forces inherent in the capillaries was already established. The results of the experiments, however, are valuable, as affording corroborative evidence that the capillary is not dependent on the general circulation.

"The experiments I offer, are all prepared by the hand of nature. They were observed, not *created*, by *artificial* methods. They are not taken from the *inferior* animals, but from *man*. This simplicity and naturalness, (so unlike the barren tortures oi vivisection—the most unnatural thing in the world, except to show the natural history of agony), will no doubt be objects of derision to those who prefer whatsoever is complex and artificial, and, who, because the vital often *coincides* or *agrees* with the *physical* law, claim everything for the latter! The facts that follow, are not referable in their main features to any merely physical, or non-vital law. They are the last acts of vitality, before yielding complete and endless submission to the laws which govern the inorganic world—the prelude to the entire extinction of life.

"If these experiments shall prove nothing more than the doctrine of the independent circulatory force of the capillary system of man, they will have accomplished more in physiology, and in pathological anatomy, than most of the experiments made on frogs, with galvanism,—dignified with the name of *Electro-Physiology, Excito-Motory-System*, &c. It is true that these vivisections (often made at the expense of the State, and by salaried professors) have, in the estimation of certain persons, so great a dignity, from the State ceremonial, as to compensate for their otherwise worthless character—experiments, as remote as *possible* from man and his healthy and morbid conditions. It is difficult to see how yellow fever subjects can be slighted, since no disease presents an equal proportion of young, vigorous, and muscular persons. A hundred cadavera from this disease, will, probably, present to the sculptor a greater per cent of model forms, than a similar number of the army itself. Plants and frogs, endosmose and galvanism, (good in their way, but withal absolutely sterile,) ought not altogether to supplant *man*, and his *natural conditions*, as subjects of medical science, human anatomy and pathology.

In the experiments on post-mortem venesection, elevated points and positions were chosen, when practicable, in order to cut off all possibility that the result might be influenced by gravity; at the same time, care was generally taken that the orifices in the arm, forehead, external jugular, &c., should be elevated but little above the level of the highest part of the body, for fear that gravity might favor the result—which it always does to a very limited extent, at the first moment, though it makes the subsequent part of the experiment far more striking and brilliant. Sometimes, the elevation was taken, by pouring water on a plank, the latter resting on the body.

All the blood, at the moment, in the *distal* end of the vein, that is, beyond the orifice, would, if raised above the level, be discharged from gravity; perhaps an ounce or more, would, in some cases, thus be lost in emptying the vein, but then, *no more could get there*, to replace the *first*, unless, by a power altogether *different from gravity*. Hence, it appears, that all the blood, with this inconsiderable exception, after the *first moment*, flowed by capillary forces—without the aid of, or rather *against*, all that is called mechanical principles—against all the chemical forces incidental to respiration—against ganglionic forces, for the ganglions were often cut away—and always, without any aid from the heart. See, and apply, yourself.

1841. Yellow Fever. A man aged 34, died,—was brought immediately, &c.; a vein in the left arm was opened—the blood flowed freely—on moving the muscles, it jetted, and upon ligation, formed an arch of about eighteen inches in diameter, as in ordinary venesection. The left jugular being opened in the usual manner, bled copiously without jetting. The abdomen and chest, were opened without delay; one of eight or ten little twigs of nearly equal size, belonging to the coronary vein of the heart, was opened upon its highest point; the blood shot out in a small, strong stream, a pint being discharged in a few minutes. The omenta and mesentery, were beautifully and forcibly distended, especially in the venous vessels. The cavas discharged from three to four pounds of blood.

1841. Yellow Fever. A man aged twenty-five, died, lying on his right side—soon after, he was placed on his back—in which position, in fifteen minutes after death, he was opened. The liver, which was very brittle, was penetrated upon its highest convex surface; in a few minutes, three and a half pounds of blood were discharged. The blood was taken up from the abdomen, in a sponge, and squeezed out into a vessel and measured. A puncture with the *finger*, would not be likely to divide any large vein, nor even in the living state, produce much hemorrhage. The capillaries of the portal system, supplied nearly all this blood, so rapidly delivered from the abdominal viscera. (At the close of life, this man had nasal hemorrhage.) Those who practice anatomical injections, know that only a few ounces of wax can be forced into the vessels of the liver.

1841. A boatman, aged 32, of extraordinary size, muscularity, and weight—dead from Yellow Fever, one hour; the jugulars being opened, the blood shot upward—in a short time, a gallon, by estimation, was discharged. [The time is not precisely noted.] At two and a half hours after death, when the dissection began, about one and a half pounds of blood, (not included in the above,) had flowed from the orifices.

1841. An Englishman, aged 37, died while I was present, of congestive, erroneously diagnosed as yellow fever: in a few minutes, the body became extremely hot; the external veins, especially in the arms, distended, as in health, after exercise—two were opened—the blood flowed rapidly, projecting about one inch; by compressing the veins, and agitating the muscles, it shot out, a foot or more, clotting firmly, as usual. The arteries were empty. The cavas discharged about four pounds of blood. An hour after death, a fine, warm, natural sweat broke out on the face, neck and chest; a strong, febrile smell emanated from the body. (After death from *solar* asphyxia, [sunstroke,] I have seen the veins of the forehead, suddenly become prominent; the face, cyanosed.)

1843. Yellow Fever. A New Yorker, aged 26:—experiments, from the third to the fifth hour after death. Room 80°; body in the axilla and stomach, 101°;* liver 102½°; thigh 100°; brain 95°; moderate rigidity. The veins of the forehead extremely prominent, to a degree rarely seen, when a man in health stoops to raise a great weight. A block placed under the back-head, raising the forehead higher than any other part of the body, caused, for a second or more, a recession of the blood, but it quickly distended the frontal vein as before. This vein was opened at its highest point on the forehead—a strong, but not a jetting stream, ran down the temple for about a quarter of an hour, discharging about twelve ounces of blood—after which, a rapid dropping, for a similar period, produced about four ounces more. The skin of the head, face and neck, which hitherto had been greatly congested, marbled, almost black, from blood in the capillaries, now became much more natural. During this venesection, an eye had been removed, and a thermometer thrust into the brain, and, also, the chest and abdomen had been opened, without any retardation of the current. It is supposed, that all the blood above the orifice in the frontal vein, subject to the flowing by gravity, did not exceed twenty drops. The orifice discharged nearly an ounce per minute—the original blood was replaced 24 times per minute—288 times in twelve minutes; or 1,440 times per hour—34,560 per day.

Here, it will be observed, is a large blood-letting, without a bandage or pressure. Can any bleeder, by this method, get half as much blood from this vein, in a living man?

It is remarkable, that all the external veins, those of the face excepted, were, in this subject, collapsed! Capillarity, in this region, alone, persisted for five hours. This man before death, had copious nasal hemorrhages.

1843. Yellow Fever. A Baltimorean, dead half an hour, was bled from the arm in the usual manner, the orifice being on a level with the highest part of the body—the blood jetted about two inches, but soon subsided to a rapid dropping. In thirty minutes, about six ounces were discharged. Contractility powerful—caloricity persisting; axilla at successive periods of 3 to 5 minutes, thus: 105°—106°—107°—107½°—etc.

1843. Yellow Fever. An Irishman, aged 33—dead two hours. The subclavian veins discharged several pounds of blood. Three hours after death, the left lobe of the liver bled profusely from an incision on its convex surface, made in taking the temperature;—an incision in the right auricle of the heart, discharged much blood. At four hours after death, the eyes still continued minutely injected. Air 61°—brain 80°; epigastrium 84°—left chest 80°—centre of the thigh 84°; contractility moderate.

1843. Yellow Fever. D., before, and for an hour after death, had minute and vivid injections of the eyes.

1842. Yellow Fever. An Englishman, dead ten minutes, was, after a few experiments on the muscles, opened. The peritoneum, omenta, mesentery, etc., had their minutest vessels, arterial and venous, distended, and bled freely when punctured. The cavas, etc., discharged, in less than an hour, from eight to ten pounds of blood, by estimation.

1842. Yellow Fever. Miss.———, aged 23; dissection, without delay. The omentum, mesentery, etc., distended with both venous and arterial

---

* For the sake of brevity, I will generally omit the experimental history of post-mortem caloricity and muscular contractility.

blood, in a manner difficult to describe, flowing freely from the smallest cut, in the higher, as well as in the lower organs. The subclavians, in a few minutes, discharged about three pounds.

1842. A German, aged 23—Yellow Fever—dead, thirty minutes. Twenty ounces of blood flowed from the external jugular, in half an hour.

1843. Yellow Fever. M.——, dead three hours.—hot—blood oozing freely from a slight puncture, at an elevated point of the shoulder or deltoidal region.

1841. Yellow Fever. An Irishman aged 35, dead 12 hours; a circular incision of the scalp, discharged in a few minutes, about six ounces of blood; incisions in the arms and legs bled freely; blood issuing from the ears; head elevated.

1841. Yellow Fever. A German aged 19, dead 30 minutes; a circular incision of the scalp, discharged forty ounces of blood in as many minutes; the head was elevated on a block.

1843. A supposed apoplectic, aged about thirty;—stout—unknown—died with apoplectic symptoms—dead one and a half hours*—heat at every two to ten minutes, in the order following:—axilla 91°, 89°; rectum 95°; thigh 92°; epigastrium and heart 94°; brain 88°; slight rigidity. Face swollen; dark red flush, as in erysipelas; pressure, as in this disease, caused the blood to recede temporarily, but it returned quickly, even in the highest parts of the head, face, etc. Incisions of the integuments of the highest part of the body, bled profusely; eyes injected for some time, say about two and a half hours after death.

I will here simply remark, without copying cases illustrative of the vascularity of the subconjunctival tissue, that real, active post-mortem injection, very different from the ecchymosed or infiltrated eye, takes place, or continues as it was before death, in considerable number of cases. As the body always rests upon the back, it disappears, of course, from gravity, as soon as the circulatory force is extinguished.

I forbear to detail cases, wherein the cadavera were not bled, though the tension of the veins, and congestion of elevated parts, often showed an existing, impulsive force,—an example of which, I observed in the person of S., two and a half hours after death from yellow fever:—1848. A slight incision on the tip of the nose, in this subject, discharged in a few minutes, four or five drachms of blood. Two days afterward, I met with another subject, which, half an hour after death, bled copiously, (as is observed, sometimes in the living body,) from the scarifications in cupping. The heart of this subject, removed from the body, gave unequivocal evidence of a circulatory capillary force, upon its exterior, as branches of the coronary vein, which were emptied by pressure, soon after became turgid again, in a manner inconsistent with gravity. The heart, of course, exercises no mechanical influence over the post-mortem capillary circulation. Nevertheless, I have experimented, in every conceivable way, upon this organ, with a view of ascertaining whether pressure might not accelerate the flow during post-mortem blood-letting. Nothing of that kind occurs.

---

* He was probably a laborer—was sent to the *Charity* Hospital, though among his rags, 45 dollars in gold and some other money, were found at the dissection.

If, however, there be blood in the arteries, it can be readily expelled, by previously opening an artery, as one of the mesenteries; I have, by a few artificial contractions, or rather compressions, spurted out to a distance, nearly half a pound of arterial blood. The removal of all the cranial, thoracic and abdominal viscera, does not arrest the capillary circulation in many regions.

The *quantity* of blood found in post-mortem examinations of fever-victims, is an important, and often a highly characteristic element in pathological anatomy, though it appears to have been almost wholly neglected; while on the other hand, nearly everything that has been said by foreign writers, of its *physical appearance*, (so far as the fevers of New Orleans are concerned) is remote from the truth. All that these writers say of the liquefaction, defibrination, putrefaction, etc., of the yellow-fever-blood, is unfounded; though, at the late periods at which examinations are usually made, such appearances are quite common. In no fever, perhaps, is the blood so natural, physically speaking, as in yellow fever. In yellow fever subjects, the *quantity of blood discharged* from the division of any great vein in the centre, as the subclavian, cava, etc., probably exceeds in a given time, the quantity from any other class of fever-subjects, owing, no doubt, chiefly to a greater quantity of blood, and a greater activity of the capillary vessels after death. I examined a subject in St. Thomas street, two hours after death from a tedious remittent, without seeing more than six ounces of blood in the brain, chest and abdomen; the most thorough dissection of every organ was made.

I will add only one more case.

1848. Serous apoplexy. The history, filling ten pages, will be slightly sketched. A stout man, aged 35, ate his breakfast as usual, and labored in the hot sun, until 9½ A. M.; the minimum of the day was 77°. He complained of indisposition, and on returning home, became insensible (as was supposed, from a stroke of the sun.) This was the opinion of his comrades. At 11 A. M. he died. The experiments began twenty-five minutes after death, and lasted one hour and thirty-five minutes before the general dissection. Contractility, too feeble to flex the fore-arm. Incipient rigidity. The thermometer remained 55 minutes in the axilla, without being removed; the first 5 minutes gave 105°;—5 m. 106½°, 5 m. 108°; 10 m. 108°; 10 m. 108°; 10 m. 108°; 10 m. 108°¼; stationary; legs crossed—the knees 108°; perineum 108°; rectum 7 m. 111°;—5 m. 111°;—15 m. 110°;—epigastrium 189¾°;—middle of the thigh 108¾°; —chest 107°.

Great distention of the veins of the head, face and neck, with red flushing of the skin, was observed. A ligature was placed on the right arm; a vein was opened as usual; about two ounces of blood jetted from the orifice, after which, the blood trickled down the arm, and in the course of two hours, discharged, in addition to the above, about twelve ounces.

The skin of the face and neck was injected, dark, livid, and somewhat mottled; there was no cadaveric hyperæmia or injection of the dependent parts; the external jugular veins were distended as if ready to burst. Greater tension I had never witnessed in glottidian œdema, nor in convulsions, nor in the last throes of parturition. The left jugular was opened, as for ordinary blood-letting, *but no bandage or pressure* was used, the head being raised, so that the orifice was nearly on a level with the breast-bone.* The blood jetted completely, without wetting the skin, forming an arch, the diameter of which, continued to extend for five minutes; at the end of 8 minutes, the arch had contracted, owing, apparently, to small clots on the margin of the orifice, and the skin having once become wet, the blood, without being materially diminished, ran down the neck, jetting occasionally on removing clots from the orifice. For about one hour the flow was copious, but, at the end of that time, was diminishing rapidly. I caught nearly three pounds at first, but as much of it did not jet out, but ran down the neck, I could only estimate the amount (which I did), at five pounds, or 80 ounces from the jugular alone. As the blood-letting progressed, the congestion and discoloration of the skin of the face, diminished.

Now it will be seen, that the orifice in the jugular did not discharge the blood as fast as the circulation replaced it—there was a surplus, because, the venous tension or jetting augmented for five minutes, and had not ceased during eight minutes. There was, as already mentioned, no bandage, or pressure. It is fair to presume, that it would be quite impossible in this way, to bleed a living man half as much, as collapse of the vein, clots, fainting, etc., would prevent it. Hence, the circulation in the veins was probably more active and persistent, than in health! Let it be supposed, that the upper or *distal* end of the jugular, contained one ounce, when opened—this being discharged, no more could replace it, only by a circulatory force. But here, the tube is filled 80 times in a few minutes. This may seem incredible from but one among hundreds of veins. A portion of this blood, however, was not derived from the distal but from the proximal end of the vein, as will be shown presently, by incontestable evidence. The explanation seems to be this: The capillaries filled the veins rapidly. The forces in the cavas, contended face to face. The right side of the heart had no outlet. The equilibrium between the ascending and descending canvas, was broken. The latter was weakened by the force or pressure turned off by the orifice, in the jugular. The pressure from below caused a retrograde movement—a very short one—towards the point where there was the least pressure, that is, the orifice in the vein.

While these experiments were being made, the cavities were opened. The heart contained but little blood.

In the early part of the experiment, particularly after the jetting of blood was changed to a stream down the neck, I observed, for a long time, that is, while the blood flowed, what then appeared to me an inexplicable phenomenon, namely, white, milky streaks, or soft flocculent masses, passing out of the orifice in the blood, rather swimming on the top of the current —a diffused drop or white flocculent cloud of this kind, was carried out once or twice every second. The subsequent dissection, fully explained this. The stomach contained nothing but water, of a sourish scent, with a few, very slender, tendinous fibres, apparently of beef, perhaps not amounting to five grains; but the quantity of chyle of a pale milky, white color, thinner than paste, in the small intestines, was very extraordinary. The

---

* This was ascertained by placing a straight or level plank on the highest points of the cadaver, and by pouring water on the plank. This body was also carefully measured, but no expansions, contractions, or elongations could be detected.

lacteals were gorged with this liquid,—the very same kind that I had seen passing from the jugular. I had, fortunately, opened the left jugular, near the place where the chyle is poured into the subclavian, so that it found an easy exit, along with the refluent current of blood, which latter, as before suggested, passed, or a part of it, in a retrograde course, proving, at once, the independent forces of the lacteal, and blood-vessels,—modified in this instance, however, by an important law of hydrodynamics, which changed their physiological directions.

This last case, (not to name others,) fruitful in physiological suggestions, shows that although life, in its popular or ultitarian sense, be completely extinct, several important sub-lives, in tissues, and several functions may survive for a considerable period, as manifested in the heat generating process, in muscular contractility, in capillary action, and in the chylous circulation. As the chylous force, so clearly proved in this case, goes to confirm, collaterally, a capillary force after general death, it may be proper to allude to the subject briefly.

Haller, the greatest physiologist of his era, ascribes the chyliferous circulation,—"first, to the attraction of capillary vessels—next to the peristaltic contraction of the intestines, and, lastly, to the alternate compressing force of the diaphragm. The attractions fills the villi, and peristaltic force empties them, and moves the chyle farther forwards. The rest of the motion seems to depend on the force of the membrane of the lacteal vessel *itself*." (Phys. DCCLXVIII.) Here, several forces, altogether imaginary or inadequate, are invoked to do almost nothing, while the lacteals, *themselves*, do all the hard work by an *inherent force*. The latter hypothesis is the most rational, or, at least, the most simple, and is therefore preferable. Certain writers of the present day, without any physical proof, or analogy, assume the existence of *molecules*, which, as they further assume, perform all the functions of nutrition and capillary circulation. This is, to say the least of it, a very obscure and undefying explanation for professed *physicists* to make. The science of physics has developed no mechanical law or force analagous to the chylous.

Müller candidly acknowledges, that the powers by which the lymph and chyle are moved, are unknown. Sis Astley Cooper, in his work on the Thoracic Duct, details experiments showing that ligation of that duct in dogs, was followed by a rupture of the receptaculum chyli, by large extravasations of chyle, and by death, in a few days. This receptacle, though weaker than the duct, "is," as he affirms, "able to bear the pressure of a column of quicksilver more than two feet in height. The force, therefore, exerted by the absorbents, must be greater than that of such a column; more especially when it is remembered that *living* parts will resist a force which would readily tear them when *dead*. The contractile powers of the absorbents are proved to be very strong—sufficient to rupture their coats." (12)

Another use of the above experiments, which I do not wish to dwell upon, is this: they completely overturn, as I conceive, the theory of Sir David Barry, namely, that the sole cause of the venous circulation, is a vacuum in the chest, caused by the act of inspiration, the atmospheric pressure causing the blood to rush into the lungs.

Endosmosis, or physical capillarity, (the physiological import of which has been already alluded to), like gravity, constantly acts upon the body and its organs; but from its known laws, it appears not to be even an auxiliary to the chylous and capillary forces. In fact, it must from its *nature oppose* them in most instances. There is, however, a use of this doctrine which has been strangely neglected, and one which I do not now intend to point out at length, namely, a use in pathological anatomy. As the vital force sinks, the endosmotic rises. Thus in a few minutes after death, *exosmose* is very apt to take place from the gall-bladder. As the bile has a peculiar color, it is easily detected in the surrounding tissues. In some dead bodies there is no exosmose from the gall-bladder, until the beginning of decomposition; but in most bodies it begins soon after, nay, probably, *before* death—certain it is that in ten minutes after the extinction of life, large tracts of the peritoneal, or subperitoneal tissues, are often infiltrated and discolored with bile that has been attracted or transmitted through the gall-bladder.

Hence, endosmose and exosmose are fundamental considerations, if not in the healthy state, they are without doubt, during, and soon after death. An example that occurred this day, (Nov. 27th,) will illustrate the point. As others often refer to plants and frogs, I may be allowed to refer to crocodiles, a far more respectable animal. One of these having perished from cold, and I, not being able to complete the dissection on the first day, threw a wet cloth over the abdomen. The gall-bladder was much distended with bile, and was not wounded. But when I returned to the dissection, the cloth was green; the whole of the bile had left the gall-bladder. The latter was shrunk almost to nothing.

Dr. Mitchell, of Virginia, from 1737 to 1741, dissected, in all, five yellow fever subjects. Some of these were kept only "two or three days" before examination! (See Cox's Med. Mus.) One of these, a negress, served the illustrious Rush, as a "clue in practice:"—"the liver." he says, "in the above mentioned slave, was turgid and plump on its outside, *but* on its *concave* surface, two-thirds of it were of a deep *black color*, and round the gall-bladder *it seemed* to be mortified and corrupted." (Inq. iii. 90.) Now the "*clue*" to this "*black* color" and this "*seeming* moritfication," is found in the transudations of bile, and other changes consequent upon the delay in the autopsy; at least, a different appearance would be extraordinary.

It would be difficult to show, in the whole circle of medical sciences, any branch more important than pathological anatomy, and, yet, is there a single work extant, which, to a greater or less extent, is not based upon examinations vitiated by physical causes or changes, endosmosis being one? Excepting a few diseases, as cancer, consumption, the lesions of which are as strong as those on the field of battle, what can be learned of color, cohesion, congestion, vascularity, turgidity, effusions, infiltrations, &c., in acute diseases, as fevers, one, two, or three days after death? Magendie, the great vivisector, of Paris, thunders in the ears of his book-making colleagues, in this wise:—"Are you not in truth convinced, that the lesions found at our autopsies are frequently produced *after* death, and that consequently the plan hitherto followed in such inquiries is *fallacious*, and can *only lead to vague information and error?*" [Lect. on the Blood.]

In this paper, I have no wish, even had I space, to make any extended applications of these researches, to physiological and morbid conditions, although, deductions, fundamentally important, are involved in the same. The scalpel is truly the greatest, and most generally successful instrument of pathological research, yet made known. But how little does it reveal in some cases of acute fever, particularly in the south, where lesions, merely secondary, or tertiary, are unfrequent? for in protracted fevers, positional lesions, doubtlessly, may form *internally* as they do *externally*. Congestions of the lungs, of the cardia and great curvature of the stomach, of the mesenteric glands, of the mucous membranes, &c., are sometimes, the result of position as much as bed-sores. I was called twenty-five miles, to see a young lady, (in Virginia,) affected with typhus, she refused all medicine; fell into a delirium for three weeks; the fever then declined, leaving a mortification of the back, from which she died three weeks after. Another young lady whom I attended daily for typhoid, during *one hundred days*, had, only a few days before death, the eruption of rose-colored, lenticular spots, described by M. Louis.

There is a constant bias, in pathological anatomy, leading to the centre, as the stomach, intestines, or some other central organs exclusively for the explanation of the cause of death, while, the circumferenee scarcely falls within the radius of pathological vision. Observe, in the early stage of yellow fever, what red flushings or capillary engorgement discolors the conjunctiva, the skin of the face, neck, and breast. As death approaches, this often is replaced by yellowness. After death, in those cases, where capillary action is most energetic, these same parts, occasionally, become much more engorged than ever,—flushed, livid, mottled, marbled, cyanosed, nay black, apparently tumid,—and this too upon parts the most elevated. Now this is as much a morbid appearance, as it would be in the cerebral or abdominal centres. This appearance is of easy explanation, upon the theory of post mortem capillary circulation. The heart discharges no blood. The capillaries deliver their blood into the veins,—these fill, as well as those large subcutaneous capillaries, that had suffered by dilation during the disease. It will be found, that as soon as the capillaries cease to act, the blood begins to gravitate; sometimes, subsiding like a water-line, or level; the higher portions of the skin, as on the cheek, forehead, tip of the nose, &c., become white. In cases where this capillary action is inconsiderable, and, in cases where it has ceased, the blood can be made to flow, and reflow, in a few seconds or minutes, by position, that is from side to side, by turning the body over; and, exactly, in those tissues where the engorgement was greatest during life, will the greatest degree of positional or gravitative engorgement be found after death. There is, however, a variety of *post mortem cyanosis*, due to capillarity, a blueness of the face, neck, &c.

Capillary hyperæmias, particularly of organs beyond sight, occur, short of softening, induration, and other phenomena characterising inflammation* in its extreme and concentrated conditions as met with in maladies less general. Now if the capillary circulation survives the general death, as it probably does, in some degree, in every case of fever, it is easy to understand, in acute hyperæmia, short of disorganization, how the capillaries, by filling up the great veins, emptying the arteries, and by altering the præ-mortem condition of their own vessels, may obliterate almost all traces of some of the most fatal morbid conditions—for inequilibrium in the circulation, is as fatal as cancer, gangrene of the lungs, or aneurisms.

---

* Professor Gross, of Louisville, in his able work on Pathological Anatomy, thus speaks concerning the *seat* of inflammation: " that this is in the capillary vessls is a fact concerning which there exists no dispute."—31

For reasons not necessary to explain, I beg leave to quote from Folio X. M. S. S., and from the Western Journal of Medicine, some dates and statements: (see my paper, Post-Mortem Researches, in the Journal; dated Jan., 1843. April No.)

November 13th, 1841;—at this date I find the following statement:—The capillary circulation in yellow fever, and other acute fevers, probably survives respiration, and the heart's action; when it ceases, cadaveric hyperæmia takes place.

In perhaps one-fourth of these dissections, the bodies were carried to the dissecting room a few minutes after death. The external veins, chiefly of the arms and neck, sometimes became distended—these being opened, the blood often flowed in a good stream—sometimes, shot out a foot or more; (that is, formed an arch having that diameter.) In some cases, by putting a ligature upon the arm, or by grasping it above the elbow, the blood was made to flow more freely, and, by moving the muscles, as the patient is wont to do in ordinary blood-letting, the blood shot out some distance. Punctures in the middle of the subclavian, discharged blood, which arose in a full stream, against gravity, two or three inches, forming, sometimes, an arch as it fell. The coronary veins of the heart, discharged blood rapidly, and with surprising force. I have observed within a few minutes after death, the flow of from twenty to thirty ounces of blood from the scalp, the head being raised. Prominence of the veins after death, is doubtlessly owing to the capillary circulation, which first fills the central, and then the external veins to excess: These dissections show conclusively the independent action of the capillaries. If capillary action exist independently of the heart, congestions, &c, may disappear, and others form during or after death, &c. Hemorrhage after death, &c. Post mortem blood-letting proves, that the heart's action is not necessary to the capillary circulation. The proofs are many, though they might have been greatly multiplied, had I taken a just view of this matter sooner—distention of the veins, copious hemorrhages, crimsoned flushings of the face, passed for nothing—not supposing it possible that the veins could discharge more blood than what they contained at the moment of death, nor that capillary life survived the extinction of the general life, as manifested in sensation, respiration, systemic circulation, &c. But, as soon as I had discovered the curious and complex laws of post-mortem contractility, the possibility of the doctrine of a post-mortem, vital capillarity, flashed across my mind. (Various co-ordinating phenomena were subsequently noticed, particularly, post-mortem caloricity, the laws of which are so peculiar as to constitute a distinct branch of thermotics.) Are not hemorrhages in yellow fever due to capillary congestion? &c. (Many other passages of similar date and import might be copied from the originals, which are open to any one.)

From the Western Journal I take the following extract, omitting the ten decided cases, each of which, proves the existence of the post-mortem capillary circulation: "The doctrine of the capillary circulation, as surviving that of the heart, and large arteries, renders tardy dissection a most fallacious guide in judging of venous congestions, vascular turgescence, organic reddenings, blanchings, &c." "I may be permitted to say, that the following facts prove that the direct action of the heart, and its indirect or suction power joined to the suction produced by respiration, are not necessary to the rapid motion of the venous blood. To say nothing of cadaveric injection and exudations, the transporting power of the capillaries might have deposited the blood in new situations, sponging it out from structures that had suffered during life from acute hyperæmia, engorging others that had been healthy, blanching the former, reddening the latter. At the right side of the heart, venous stagnation probably begins, its vessels being wholly passive. The capillary power is soon neutralized by an opposing mechanical force, or ended by an entire cessation of vital action."

I make these extracts for the consideration of honest Æsculapians, (these are not few) who are willing to give every man his due. If any one has ever before established the independent action of the capillary circulation in the living or dead human subject, if any one has applied this discovery, a fruitful one, in explanation of pathology and morbid anatomy, I am ignorant of the fact."

